# Supplementary material for: Nicotinamide mononucleotide enhances anti-tumor effect by resetting macrophages toward the inflammatory M1-like phenotype
Source: Mol Ther Oncol. 2026 Apr 28;34(2):201221. doi: 10.1016/j.omton.2026.201221 (PMC13195322; doi:10.1016/j.omton.2026.201221)
Supplement: Document S1. Figures S1–S3 and Table S1 [file mmc1.pdf]

**OMTON, Volume *34***

## Supplemental information

# Nicotinamide mononucleotide enhances anti-tumor effect by resetting macrophages toward the inflammatory M1-like phenotype

**Haoran Xu, Marcus Chun Tao Wan, Chelsey Chi Ching Wong, Siqi Qin, Yang Wen, Jun Wang, and Zhiwei Chen**

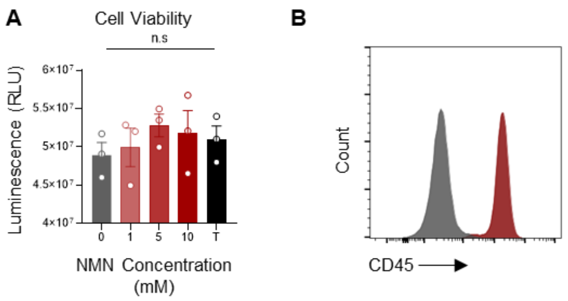


**Figure S1. Experimental setup for huPBMC-tumor co-culture system.** (**A**) The viability of tumor cells attached to the plate was determined by CellTiter-Glo® luminescent cell viability assay (Promega) at 24 hours post-co-culture with immune cells. The co-culture assay was set up following the same conditions as in Figure 1, and tumor cell only (T) served as the positive control (100% live cells) for viability measurement. (**B**) Cells harvested from the supernatant of three 10mM NMN-treated huPBMC-tumor coculture samples were combined and proceeded to the FACS for cell type analysis. All cells were divided into two samples for isotype control staining (grey) and CD45$^{+}$ staining (red). Statistics in **A** were generated by one-way ANOVA with post-hoc correction followed by multiple comparisons.

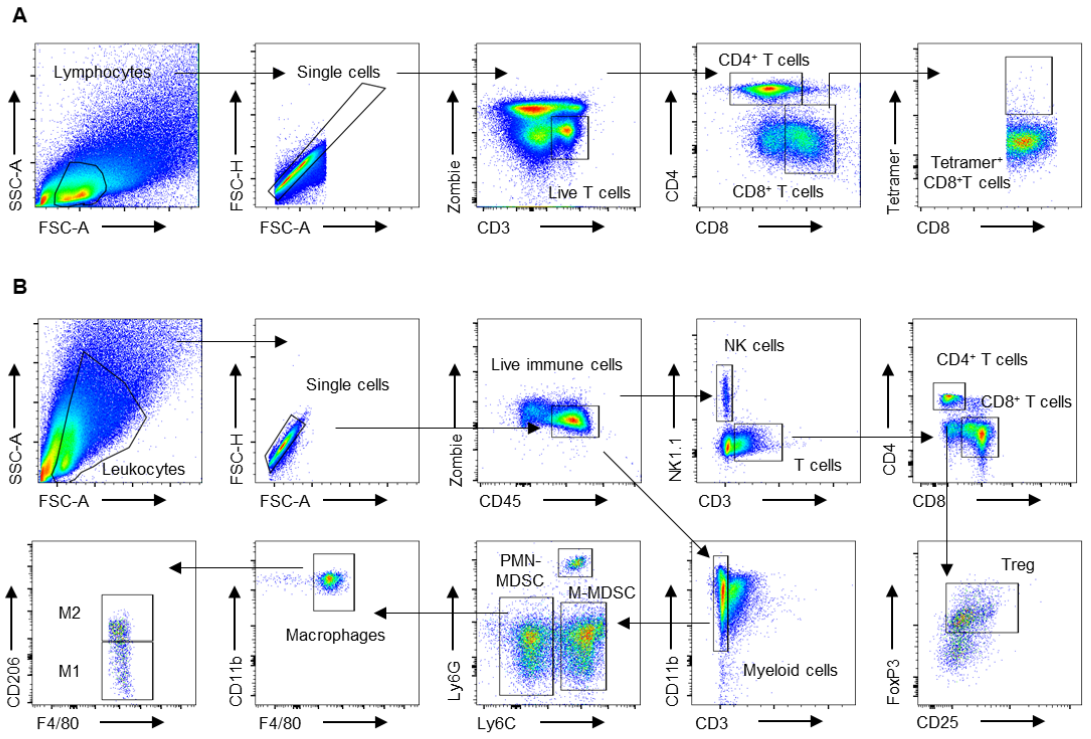


**Figure S2. FACS Gating strategy.** (**A**) Gating strategy for tumor-specific CD8$^{+}$ T cells in spleen. (**B**) Gating strategy for intratumor immune cells.

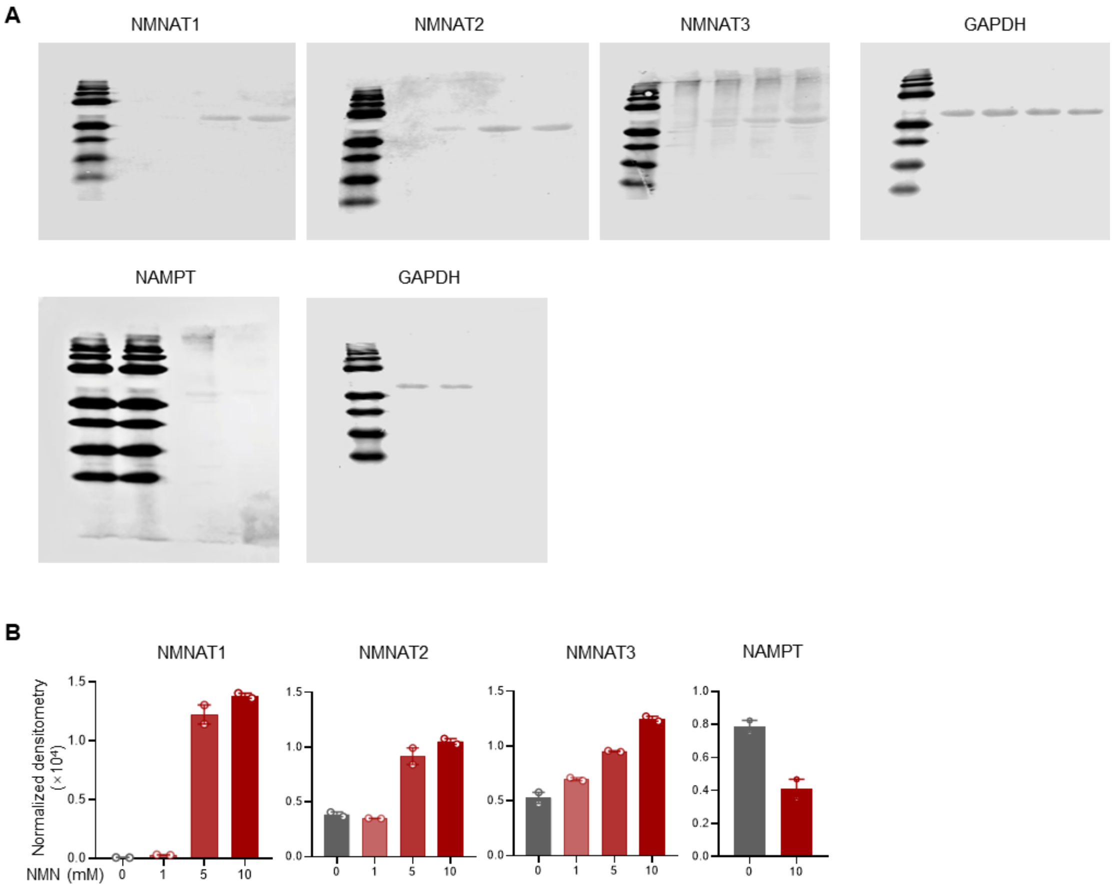


**Figure S3. Original Western Blot data and densitometry quantification.** (**A**) Matched original Western Blot data of Figure 1E and F (**B**) Densitometry quantification results from two independent experiments.

**Table S1. List of primers for RT-qPCR.**

| Primer pair | Forward/ Reverse | Target sequence (5' → 3') |
|---|---|---|
| CD38 (mouse) | Forward | TCTCTAGGAAAGCCCAGATCG |
| | Reverse | AGAAAAGTGCTTCGTGGTAGG |
| GAPDH (human) | Forward | GGAGCGAGATCCCTCCAAAAT |
| | Reverse | GGCTGTTGTCATACTTCTCATGG |
| GAPDH (mouse) | Forward | AGGTCGGTGTGAACGGATTTG |
| | Reverse | GGGGTCGTTGATGGCAACA |
| IL-12 β (mouse) | Forward | GTCCTCAGAAGCTAACCATCTCC |
| | Reverse | CCAGAGCCTATGACTCCATGTC |
| NAMPT (human) | Forward | CGGCAGAAGCCGAGTTCAA |
| | Reverse | GCTTGTGTTGGGTGGATATTGTT |
| NMNAT1 (human) | Forward | TCTCCTTGCTTGTGGTTCATTC |
| | Reverse | TGACAACTGTGTACCTTCCTGTT |
| NMNAT2 (human) | Forward | TGTCCACGACTCCTATGGAAA |
| | Reverse | GTCCGATCACAGGTGTCATGG |
| NMNAT3 (human) | Forward | GAGTAGGTCACGACCCAAAAG |
| | Reverse | TCGCCTGATGTATGTGGCAC |
| Slc12a8 (human) | Forward | CTGGTGTCCTTCGTCATCCTG |
| | Reverse | CACCTGCAACACACTGTCCA |
| TNFa (mouse) | Forward | CAGGCGGTGCCTATGTCTC |
| | Reverse | CGATCACCCCGAAGTTCAGTAG |
