# Supplementary material for: Nicotinamide mononucleotide enhances anti-tumor effect by resetting macrophages toward the inflammatory M1-like phenotype
Source: Mol Ther Oncol. 2026 Apr 28;34(2):201221. doi: 10.1016/j.omton.2026.201221 (PMC13195322; doi:10.1016/j.omton.2026.201221)
Supplement: Document S2. Article plus supplemental information [file mmc2.pdf]

Molecular Therapy
Oncology
Brief report

Nicotinamide mononucleotide enhances anti-tumor effect by resetting macrophages toward the inflammatory M1-like phenotype

Haoran Xu,[1,2,3,4,6] Marcus Chun Tao Wan,[1,2,6] Chelsey Chi Ching Wong,[1,2] Siqi Qin,[1,2] Yang Wen,[1,2,3] Jun Wang,[5] and Zhiwei Chen[1,2,3,4]

[1]AIDS Institute, School of Clinical Medicine, Li Ka Shing Faculty of Medicine, The University of Hong Kong, 21 Sassoon Road, Pokfulam, Hong Kong SAR, People's Republic of China; [2]Department of Microbiology and Pandemic Research Alliance Unit, School of Clinical Medicine, Li Ka Shing Faculty of Medicine, The University of Hong Kong, 21 Sassoon Road, Pokfulam, Hong Kong SAR, People's Republic of China; [3]State Key Laboratory of Emerging Infectious Diseases, The University of Hong Kong, 21 Sassoon Road, Pokfulam, Hong Kong SAR, People's Republic of China; [4]Center for Virology, Vaccinology and Therapeutics, Hong Kong Science and Technology Park, Hong Kong SAR, People's Republic of China; [5]GeneHarbor (Hong Kong) Biotechnologies Ltd., Hong Kong Science and Technology Park, Hong Kong SAR, People's Republic of China

Nicotinamide mononucleotide (NMN) supplementation has shown clinical benefits by regulating metabolic activities in the energy production process. Its protective effect and underlying immune regulatory mechanisms against tumor progression are still poorly understood. Here, we found that the high-dose NMN treatment could alter the level of several key NAD^+ metabolic enzymes in human immune cells. Moreover, high-dose NMN treatment exhibited comparable anti-tumor efficacy as the PD-1 blockade in the murine mesothelioma challenge model. Subsequent immune profiling in both secondary lymphoid organ and tumor demonstrated that, rather than modulating T cell and NK cell responses, high-dose NMN treatment could reset tumor-associated macrophages toward the inflammatory M1-like phenotype compared with PD-1 blockade or non-treated subjects. These results provided a better understanding of NMN's regulatory effect on immune cells and suggested an alternative strategy of cancer immunotherapy.

INTRODUCTION

Immune checkpoint blockade (ICB) is the most promising antitumor immunotherapy in recent decades, and programmed cell death protein 1 (PD-1) pathway blockade serves as one of the most widely applied therapies in this field.[1,2] By disrupting the binding between PD-1 and its immune-suppressing ligands (PD-L1), PD-1 blockade can rescue the exhausted cytotoxic T cells and restore the T cell-mediated antitumor immunity. However, many types of cancers in humans have been reported with high levels of PD-L1 expression, and PD-L1 is also found on different myeloid cells in the tumor microenvironment, which may further impair the effectiveness of anti-PD1 immunotherapy on tumor-infiltrating lymphocytes (TILs).[3,4] To resolve the current limitations of PD-1 blockade and other immunotherapies, alternative strategies targeting immune pathways in both innate and adaptive immunity are still being extensively explored.

As an intermediate nucleotide in the nicotinamide adenine dinucleotide (NAD^+) salvage pathway, nicotinamide mononucleotide (NMN) is essential to systemic NAD^+ synthesis and energy production.[5] Previous research primarily focused on its anti-aging potential by increasing systemic NAD^+ levels, enhancing mitochondrial function, and other cellular metabolic mechanisms.[6] Emerging recent research has demonstrated the role of NMN and its metabolites in regulating the immune system against infectious diseases and cancer through activating certain NAD-consuming factors such as sirtuins, ADP-ribose polymerases (PARPs), and CD38.[5,7] NMN has also been proven to show tumor inhibition effects at 100 mM against lung adenocarcinoma.[8] A possible underlying mechanism suggested that high-dose NMN intervention regulates the intracellular level of reactive oxygen species (ROS) together with lipid peroxidation. Subsequent increased consumption of glutathione and accumulation of malonaldehyde with mitochondrial ferrous ions induce antitumor ferroptosis.[8,9] Although NMN treatment exhibits great potential against cancer progression, its regulatory effect on immune cell metabolism within the tumor microenvironment is still poorly understood, and further investigation is warranted.

In this study, we focused on the immune-modulating effect of NMN in both human and murine immune cells. We first demonstrated that NMN treatment could modulate the expression of several key metabolic enzymes in the NAD^+ salvage pathway in immune cells within the tumor microenvironment. Subsequent *in vivo* tumor challenge confirmed that the enhanced NAD^+ synthesis was associated with

Received 6 November 2025; accepted 24 April 2026;
https://doi.org/10.1016/j.omton.2026.201221.

[6]These authors contributed equally

Correspondence: Zhiwei Chen, AIDS Institute, School of Clinical Medicine, Li Ka Shing Faculty of Medicine, The University of Hong Kong, 21 Sassoon Road, Pokfulam, Hong Kong SAR, People's Republic of China.
E-mail: zchenai@hku.hk

© 2026 The Author(s). Published by Elsevier Inc. on behalf of The American Society of Gene and Cell Therapy. This is an open access article under the CC BY license (http://creativecommons.org/licenses/by/4.0/).

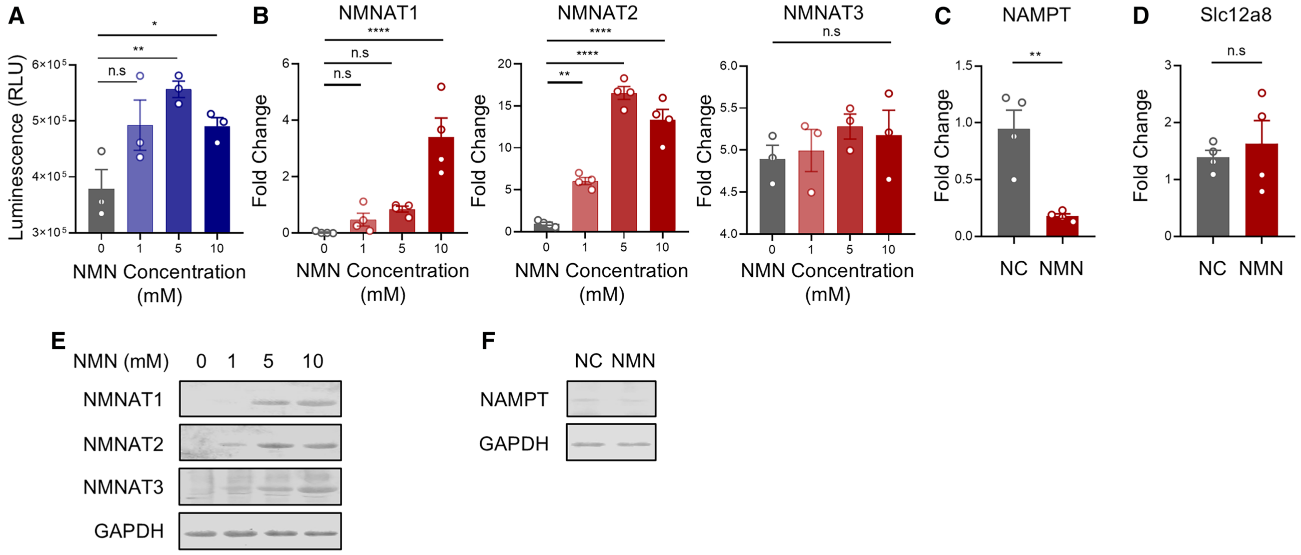

Figure 1. NMN treatment regulated the expression of metabolic enzymes in the NAD^+ salvage pathway in human immune cells
(A) The intracellular NAD^+ level in human PBMCs collected from the huPBMC-tumor co-culture was detected using NAD/NADH-Glo assay. (B) Fold changes in mRNA levels of NMNAT 1 to 3 in immune cells harvested from supernatant upon 24 h post NMN treatment at 0, 1, 5, and 10 mM concentration. All the fold changes were normalized with the non-treated group. (C) Fold changes in mRNA levels of NAMPT in immune cells harvested from supernatant in the group of 10 mM NMN treatment and non-treated samples (NC), respectively, at 24 h post co-culture. (D) Fold changes in mRNA levels of Slc12a8 in immune cells harvested from supernatant in the group of 10 mM NMN treatment and non-treated samples (NC), respectively, at 24 h post co-culture. (E) Expression of NMNAT 1 to 3 in immune cells harvested from supernatant upon 24 h post NMN treatment at 0, 1, 5, and 10mM concentration was detected by western blot. (F) Expression of NAMPT in immune cells harvested from supernatant in the group of 10 mM NMN treatment (NMN) and non-treated samples (NC), respectively, at 24 h post co-culture. The group size of A was $N = 3$, and $N = 4$ was adopted in the rest of the experiments. (E) and (F) are representative results from two independent experiments. Statistics in (A) and (B) were generated by one-way ANOVA with post hoc correction followed by multiple comparisons. Statistics in C and D were generated by the two-tailed Student *t* test.

better protective efficacy against mesothelioma. Results of immune profiling from cells isolated in the spleen and tumor suggested that rather than T cell- and natural killer (NK) cell-related immune responses, the antitumor response elicited by high-dose NMN treatment relied on resetting tumor-associated macrophages (TAMs) toward the inflammatory M1-like phenotype. Our preliminary findings gave new insight into the metabolic regulatory effect of NMN in immune cells and revealed an alternative mechanism underlying the enhanced mesothelioma protection by high-dose NMN treatment.

RESULTS

High-dose NMN treatment altered the mRNA level of NMN-related metabolic enzymes in human PBMCs

To evaluate the potential metabolic modulating effect of NMN on human immune cells within the tumor microenvironment, we set up a human (hu) peripheral blood mononuclear cell (PBMC, huPBMC)-tumor cell *in vitro* co-culture system. We first confirmed that most cells harvested from the supernatant exhibited the phenotype of immune cells, and that downstream detection would not be significantly affected by tumor cells attached to the plates. (Figure S1). The effective uptake and metabolism of supplemented NMN by human PBMCs within the co-culture system were then verified (Figure 1A). We subsequently measured the changes in mRNA level of different key metabolic enzymes involved in the NAD^+ salvage pathway, including nicotinamide mononucleotide adenylyltransferases (NMNATs) and nicotinamide phosphoribosyltransferase (NAMPT). (Figures 1B and 1C). In the co-culture system, NMN treatment induced a significant increase in the mRNA levels of NAD^+ synthesis-associated enzymes. This augmentation in mRNA levels of NAD^+ synthesis-associated enzymes was also dose-dependent (Figure 1B). On the contrary, the mRNA level of NAMPT, an intracellular NMN synthesis-related enzyme, was observed to decrease at the high-dose NMN treatment condition (Figure 1C). We did not observe any changes in Slc12a8, the direct cellular transporter for NMN, upon high-dose treatment (Figure 1D). Subsequent western blot further confirmed the consistent changes in protein level of the above NAD^+ synthesis-associated enzymes upon NMN treatment (Figures 1E and 1F). Collectively, the high-dose NMN treatment could alter the expression levels of NMN-related metabolic enzymes in human PBMCs, which also indicated a potential metabolic modulating effect on various immune cells in the tumor microenvironment.

NMN treatment reset macrophages toward the inflammatory phenotype and show a comparable inhibition effect to PD-1 blockade against mesothelioma progression

We next sought to investigate whether the enhanced NAD^+ salvage pathway in peripheral immune cells is associated with improved anti-tumor effect. We first confirmed that the high-dose NMN treatment was safe by measuring body weight changes during a 14-day

treatment (Figure 2A). Murine AB1 mesothelioma cell line expressing HIV-1 Gag model antigen was introduced to further characterize both non-specific innate immunity and antigen-specific T cell responses in the tumor microenvironment.[10] PD-1 blockade was previously reported to inhibit the mesothelioma progression,[11–13] and therefore, was involved as the control to evaluate the anti-tumor effect mediated by NMN treatment (Figure 2B). Surprisingly, we found that high-dose NMN treatment exhibited a comparable inhibition effect against mesothelioma progression (Figures 2C–2E). We further profiled the immune cells in the spleen and tumor isolated from the mesothelioma-challenged mice to elucidate the primary component mediating the anti-mesothelioma effect (Figure S2). The frequency of systemic tumor-specific $CD8^+$ T cells was significantly higher in the PD-1 blockade group, which is consistent with the previous finding.[14,15] However, NMN treatment did not elicit a similar effect on tumor-specific T cells (Figure 2F). Meanwhile, no significant difference was observed in the frequencies of intra-tumor NK cells ($CD3^-$ $NK1.1^+$), Treg cells ($CD4^+$ $CD25^+$ $Foxp3^+$), polymorphonuclear myeloid-derived suppressor cells (PMN-MDSCs) ($CD11b^+$ $Ly6G^+$), and M-MDSCs ($CD11b^+$ $Ly6C^+$) after NMN treatment (Figure 2F). Apart from the above immune subsets, a significant phenotype change of M1-/M2-like TAM ($CD206^{-/+}$ $F4/80^+$ $CD11b^+$) was observed in the NMN treatment group only (Figures 2G and 2H). The frequency of inflammatory M1-like macrophages was significantly increased after NMN treatment, while the frequency of immunosuppressive M2-like macrophages was significantly decreased compared with other groups (Figures 2G and 2H). To further demonstrate that high-dose NMN treatment enhanced the anti-tumor function of M1-like macrophages, macrophages were depleted systemically using Colony Stimulating Factor 1 Receptor (CSF1R) inhibitor in NMN-treated mice during mesothelioma tumor challenge (Figure 2I). Depletion of macrophages significantly impaired the high-dose NMN-mediated tumor protection, resulting in a similar tumor growth rate compared with untreated mice (Figure 2J). These data collectively indicated that the reshaping of the M1-like/M2-like phenotype of TAM might contribute to the enhanced anti-mesothelioma effect by NMN treatment.

NMN treatment promoted the activation and anti-tumor cytokine secretion of systemic macrophages

To further confirm that the phenotype change in TAM was directly associated with enhanced anti-tumor function, total macrophages were isolated from the spleen in AB1 subcutaneous (s.c.)-challenged mice and subsequently co-cultured with tumor cells with or without NMN supplementation. Total T cells were also isolated and subjected to the same procedure to elucidate consistency with the *in vivo* observation (Figures 3A–3C). Compared to the non-treated group, the mRNA levels of the anti-tumor cytokine TNF-α were significantly upregulated in macrophages (Figure 3D).[16] Meanwhile, the mRNA levels of the activation markers IL-12β and CD38 were also upregulated at 24 h post NMN treatment (Figure 3D).[17,18] No significant changes in the mRNA levels of TNF-α were observed but a slight increase in the activation markers of IL-12β and CD38 was observed in T cells (Figure 3E). To further confirm this finding *in vivo*, macrophages and T cells were isolated from the tumor in mesothelioma-challenged mice, with or without NMN treatment, for fluorescence-activated cell sorting (FACS) analysis. Consistent trends of protein-level changes in TNF-α, IL-12β, and CD38 were also observed in macrophages (Figure 3F), rather than T cells (Figure 3G), from high-dose NMN-treated mice at the tumor challenge endpoint. Moreover, a higher frequency of inflammatory cytokine IFN-γ was observed in macrophages from the NMN-treated group but no difference in granzyme B was found (Figures 3F and 3G). In summary, high-dose NMN treatment promoted the activation and secretion of anti-tumor cytokines in macrophages, which contribute to enhanced tumor inhibition effect.

DISCUSSION

Recent studies suggested that supplementation of intermediates in the NAD^+ biosynthesis process, including nicotinamide riboside (NR) and NMN, show clinical therapeutic benefits.[6] NAD^+ is both a coenzyme for hydride-transfer enzymes and a substrate for NAD^+-consuming enzymes, which include ADP-ribose transferases, poly-ADP-ribose polymerases, cADP-ribose synthases, and sirtuins.[5] The maintenance of a high NAD/NADH ratio in mitochondria is proven to be essential for ATP mitochondrial production.[19] However, limited research focused on the modulation of NAD^+ metabolism and its biosynthetic intermediates in the immune system. We firstly evaluated the changes in several key metabolic enzymes in the NAD^+ salvage pathway in immune cells after NMN treatment. NAMPT initiates NMN biosynthesis from nicotinamide, and NMNAT 1 to 3 serve as the key enzymes to synthesize NAD^+ from NMN.[20] According to our results, high-dose NMN treatment induced a significant increase in the expression of NMNATs 1 to 3 and a decrease in NAMPT (Figure 1). Previous research observed a considerable increase in the NMNATs after NMN supplementation in Parkinson's disease (PD) cell models.[21] Although the detailed molecular mechanism still requires future investigation, we extended the similar finding to the immune cells within the tumor microenvironment, that the concentration of intermediates affects the enzyme expression in the catalytic cycle of the NAD^+ salvage pathway.

Current research on the antitumor effects of NMN primarily focuses on its mitochondrial metabolism modulation in tumor cells, which directly affects the physiological activity of tumor cells.[6] The general regulatory effect on the NAD^+ salvage pathway in immune cells by NMN supplementation may also indirectly mediate the enhanced antitumor response. With the observation that high-dose NMN treatment could achieve comparable inhibition efficacy against mesothelioma, we conducted a comprehensive profiling of multiple immune subsets in both the secondary lymphoid organ and tumor (Figures 2F and 2G). We identified a significant change in TAMs toward the inflammatory M1-like phenotype induced by the high-dose NMN only (Figures 2G and 2H). Previous reports have emphasized that the polarization and inflammatory cytokine production capacity of M1-like macrophages are positively correlated with enhanced tumor protection.[22] Results from both *in vitro* and *in vivo* functional assays were consistent with previous research and served as a possible mechanism

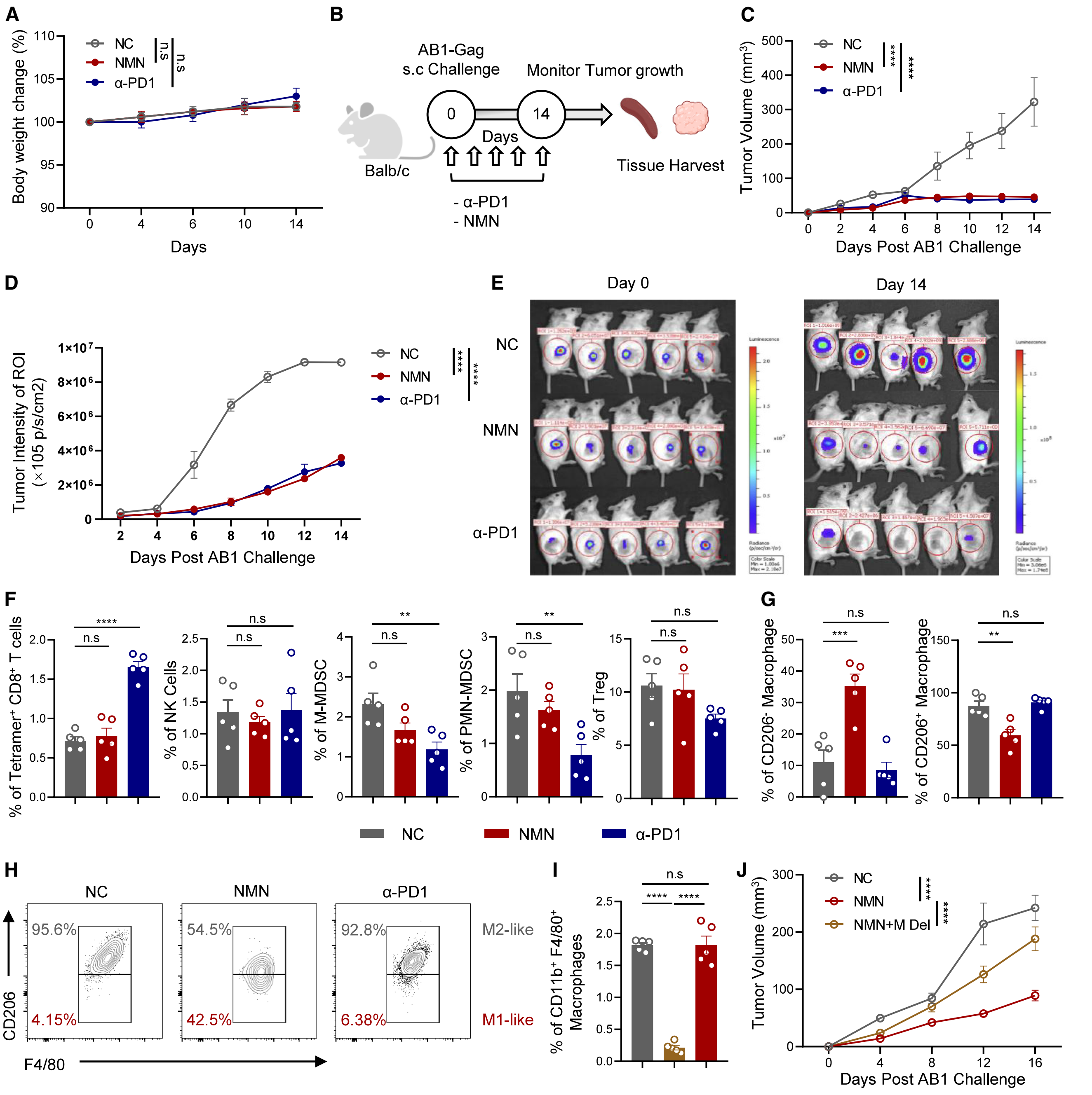

Figure 2. High-dose NMN treatment reset macrophages toward the inflammatory M1-like phenotype and enhanced the protective efficacy against mesothelioma

(A) The relative changes to the initial body weight of naive mice receiving different treatments: NMN only (NMN), anti-PD1 (α-PD1), and non-treated (NC). (B) Tumor challenge schemes. Technical replicates of $N = 5$ were adopted for each group. (C) Tumor growth kinetics measured by tumor size. (D) Tumor growth kinetics measured by luminescence intensity. (E) Tumor luminescence intensity among different groups at the endpoint. (F) Frequencies of Gag tetramer-positive $CD8^+$ T cells isolated from the spleen among different groups. Frequencies of PMN-MDSC, M-MDSC, NK, and Treg cells were quantified in the tumor among different groups. (G) Frequencies of M1- and M2-like macrophages among different groups. (H) Density plots representing $CD206^-$ M1-like macrophages and $CD206^+$ M2-like macrophages were gated in $CD3^-$ $Ly6C^-$ $F4/80^+$ $CD11b^+$ live cells. (I) Frequencies of $Ly6C^-$ $F4/80^+$ $CD11b^+$ macrophages in total live $CD45^+$ immune cells in the tumor among different groups at day 16 post tumor challenge. (J) Tumor growth kinetics measured by tumor size among NMN treatment only (NMN), NMN treatment plus macrophage depletion (NMN+M Del), and non-treated (NC). Biological replicates of $N = 5$ were adopted for each group in (I) and (J). two-way ANOVA was applied for (A), (C), (D), and (J). Statistics in (F), (G), and (I) were generated by one-way ANOVA with post hoc correction followed by multiple comparisons. (C and D) Data from one representative experiment of two independent experiments was shown.

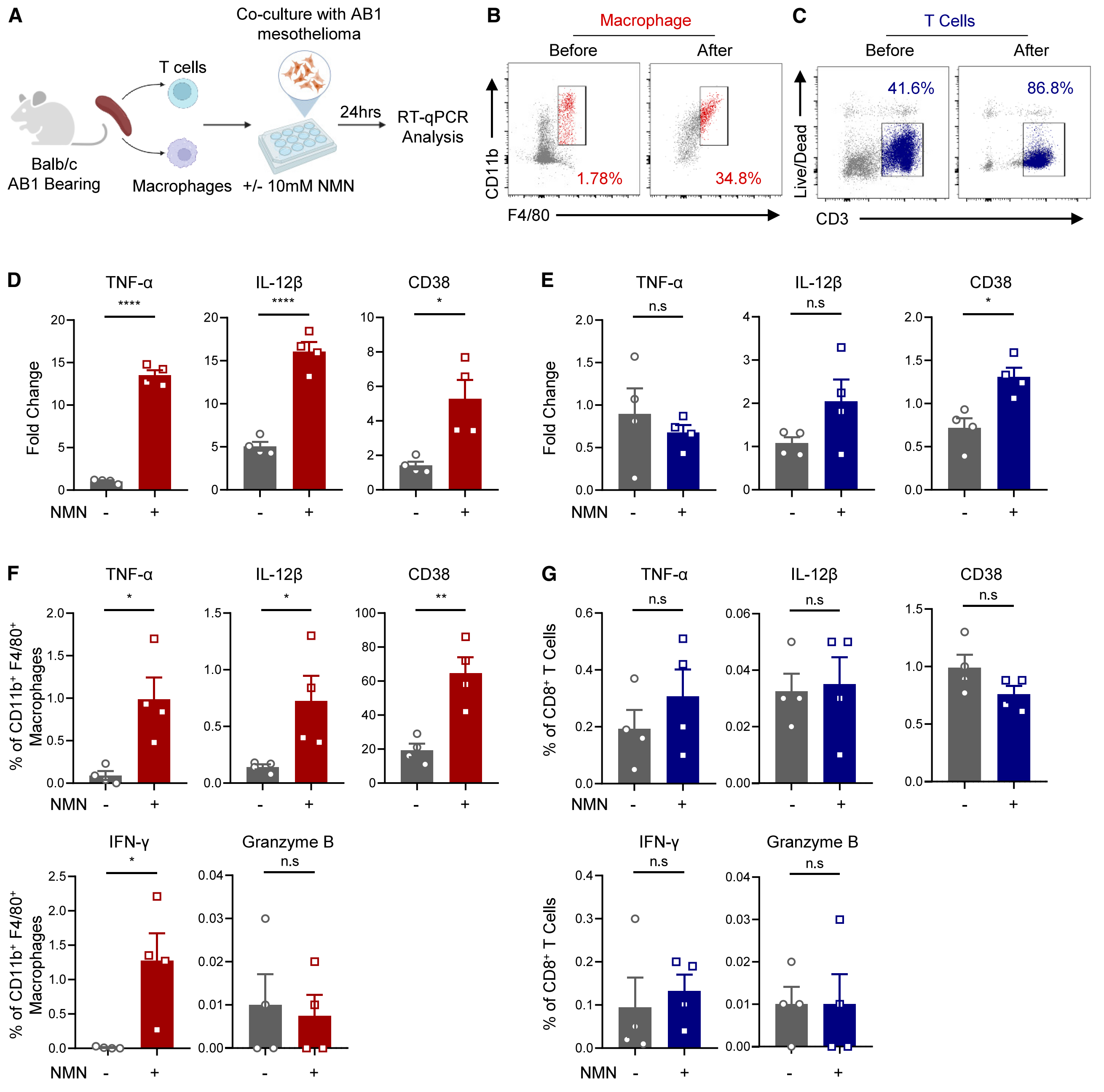

Figure 3. NMN treatment enhanced the activation and cytokine secretion of macrophages

(A) Experiment schemes. Four technical replicates were set for each group. Macrophage (B) and T cell (C) purification efficacy was verified by FACS analysis using samples collected before and after isolation. (D) Fold changes in mRNA levels of TNF-α, IL-12β, and CD38 in macrophages collected from supernatant at 24 h post-co-culture. (E) Fold changes in mRNA levels of TNF-α, IL-12β, and CD38 in T cells collected from supernatant at 24 h post-co-culture. (F and G) Cells collected from tumor in mice treated with or without NMN in Figure 2J at the endpoint were subjected to the FACS analysis. (F) Frequencies of TNF-α, IL-12β, CD38, IFN-γ, and granzyme B expression in macrophages. (G) Frequencies of TNF-α, IL-12β, CD38, IFN-γ, and granzyme B expression in $CD8^+$ T cells. Statistics for (D–G) were generated by the two-tailed Student *t* test.

for the improved mesothelioma protection (Figure 3). Additional studies also revealed that immune checkpoints, including PD-L1 and PD-1 are expressed in TAMs.[4] The tumor immune evasion to current ICB may also induce dysfunctional M1-like macrophage-mediated responses. Overall, our work preliminarily demonstrated an alternative immune regulatory approach to ICB.

MATERIALS AND METHODS

Mice

Male and female 6–8 weeks old BALB/c mice were obtained from the University of Hong Kong Center for comparative medicine research. All animals were housed in biosafety level 2 pathogen-free conditions. All experiments were approved by the Committee on the Use of Live Animals in Teaching and Research, The University of Hong Kong.

Human blood samples

Healthy human blood buffy coats were retrieved from the Hong Kong Red Cross Blood Transfusion Service under prior informed and signed consent. The median age of the donor is 31, with Hepatitis B Virus/Hepatitis C Virus negative test results. huPBMCs are subsequently isolated by density gradient centrifugation with Lymphoprep (Axis-Shield) for further processing in *in vitro* co-culture experiments. The use of buffy coats received ethics approval from the Institutional Review Board of the University of Hong Kong/ Hospital Authority Hong Kong West Cluster #UW13-476.

Cell lines

Human lung adenocarcinoma A549 cell line was retrieved from the American Type Culture Collection (ATCC) and maintained in DMEM with 10% fetal bovine serum (FBS), 100 U/mL penicillin, and 100 μg/mL streptomycin sulfate (1% P/S). Murine mesothelioma AB1 cell line expressing HIV-Gag and luciferase (AB1-Gag) was developed in-house and cultured in DMEM supplemented with 10% FBS, 1% P/S, and 1 μg/mL puromycin. All cells were maintained and stored at 37°C, 21% O_2, and 5% CO_2. Routine biweekly mycoplasma PCR detection was also conducted before usage.

Tumor-immune cell co-culture system

For the A549-huPBMCs co-culture system, A549 cells were prepared at a density of 1×10^5 cells per well in the 12-well plate. Isolated huPBMCs were subsequently added at a ratio of 10:1 to seeded A549 cells. NMN at different concentrations were added to the co-culture accordingly and treated for an extra 24 h. The CellTiter-Glo luminescent cell viability assay (Promega) was applied to the measurement of the viability of tumor cells attached to the plate. Cells harvested from the supernatant were processed for FACS to analyze the cell type. The above two assays collectively confirmed that cells isolated from the supernatant only consisted of immune cells in the co-culture system. For the AB1-T cell and AB1-macrophage co-culture system, AB1-Gag cells were prepared at a density of 1×10^5 cells per well in the 12-well plate. T cells were isolated from mouse spleen using mouse pan T cell isolation kit (Miltenyl Biotec, Cat#130-095-130), while macrophages were prepared using mouse macrophage isolation kit (Miltenyl Biotec, Cat#130-110-434), respectively. Isolated mouse T cells or macrophages were subsequently added at a ratio of 10:1 to seeded AB1-Gag cells separately. NMN at different concentrations were added to the co-culture accordingly and treated for an extra 24 h. Supernatants from the co-culture system were collected at 24 h post co-culture.

For the detection of intracellular NAD level, supernatant from the co-culture system was harvested and centrifuged to collect the immune cells. Collected immune cells were lysed for measuring relative luciferase units (RLUs) with NAD/NADH-Glo Assay (Promega).

For the detection of different enzymes in the NAD^+ salvage pathway, supernatant from co-culture system was collected and centrifuged to harvest the immune cells, and subsequently proceeded to RNA extraction and real-time quantitative PCR (RT-qPCR).

RT-qPCR

RNA was extracted from cells in the supernatant using RNeasy Mini Kit (QIAGEN, Cat#74106). cDNA was prepared from 1 μg of RNA using PrimeScript II 1st Strand cDNA Synthesis Kit (Takara). One-Step TB Green PrimeScript reverse-transcription PCR (RT-PCR) Kit II (Takara) was used for the quantitative real-time PCR assay. Quantitative real-time PCR assay was performed on ViiA 7 Real-Time PCR System (Thermo Scientific). The primers involved in RT-qPCR assay were listed in Table S1.

In vivo AB1-Gag tumor challenge

In the tumor challenge assays, 8–10 weeks BALB/c mice were used . About 1×10^6 AB1-Gag cells were s.c. injected into the mice at the left flank regions. All the treatments were started at the same time as the tumor inoculation. Tumor-inoculated mice received PBS, mouse anti-PD1 (Clone: J43, InVivoMAb, Cat# BE0033-2-100MG) at the 200 mg/kg dosage, and NMN (beta-NMN from Geneharbor) at the 300 mg/kg dosage, respectively. The high dose of NMN treatment for mice is equivalent to 25 mg/kg for human as the clinical high-dose setting.[7] The dose for anti-PD1 treatment followed the previous research study.[23] All the treatments were inoculated intraperitoneally (i.p.) twice per week and lasted until the tumor challenge endpoint. Tumor size was measured every 2 days using the caliper. Luciferase-expressing tumor growth kinetics was screened in the IVIS Spectrum System (PerkinElmer), and luminescent intensity was measured within the regions of interest (ROI). Bioluminescence intensity within the ROI was presented as photons/s/cm^2/sr. Acquired data were analyzed with Live Imaging software (v.4.0; PerkinElmer).

Macrophage depletion assays

Macrophage depletion started 2 days before tumor planting by i.p. injection of 200 μg anti-mouse CSF1R (CD115) (BioXcell) in 200 μL PBS. Subsequent macrophage depletion was performed twice per week together with the NMN treatment. The depletion efficacy was confirmed by FACS analysis of cells isolated from the tumor at the tumor challenge endpoint.

Flow cytometry and antibodies

Following anti-mouse antibodies were purchased and used at 1:100 dilution: Alexa Fluor 647 anti-mouse FOXP3 (Clone: MF-14, Cat#126408), APC anti-mouse/human CD11b (Clone: M1-like/70, Cat# 101212), APC/Cyanine7 anti-mouse CD45 (Clone: 30-F11, Cat#103116), APC/Cyanine7 anti-mouse CD8a (Clone: 53–6.7, Cat#

100714), Brilliant Violet 421 anti-mouse F4/80 (Clone: BM8, Cat#123137), Brilliant Violet 785 anti-mouse CD3 (Clone: 17A2, Cat# 100232), Brilliant Violet 785 anti-mouse Ly-6C (Clone: HK1.4, Cat#128041), PE anti-mouse Ly-6G (Clone: 1A8, Cat# 551461), PE/Cyanine7 anti-mouse CD25 (Clone: PC61.5, Cat# 25-0251-82), PerCP/Cyanine5.5 anti-mouse NK-1.1 (Clone: PK136, Cat# 108728), Pacific Blue anti-mouse CD4 (Clone: RM 4–5, Cat# 48-0042-82), Zombie Aqua Fixable Viability Kit (BioLegend, Cat# 423102). For tetramer staining assay, PE-conjugated H-2Kd HIV-1 Gag AMQMLKETI (AI9) MHC class I tetramer (MBL) was cultured with cells isolated from the mouse spleen at 1:50 dilution at room temperature for 15 min, and other surface marker antibodies were added at designated dilution and stained at 4°C for 30 min. Stained samples were acquired with BD FACS Aria III cell sorter (BD Biosciences), and the data were analyzed by FlowJo v.10.

The following antibodies were purchased and used for western blot at 1:500 dilution: anti-Nmnat1/NMNAT antibody (OTI1F7, abcam), Anti-NMNAT2 antibody (2E4, abcam), Anti-NMNAT3 antibody (abcam), NAMPT Monoclonal Antibody (Invitrogen).

Statistical analysis

Statistical analysis was performed using GraphPad Prism 8.0. Designated statistical methods applied to different analyses were listed in the figure legends. All error bars indicate SEM. A p value <0.05 was considered significant, n.s., $p > 0.05$; *, $p < 0.05$; **, $p < 0.01$; ***, $p < 0.001$; ****, $p < 0.0001$. The number of research subjects in each group and the specific details of statistical tests are reported in the figure legends.

DATA AND CODE AVAILABILITY

All relevant data within the manuscript are available in the main text or the supplemental information. Other raw data/materials used in this study are available upon request to the corresponding author.

ACKNOWLEDGMENTS

We would like to thank Prof. Honglin Chen and Dr. Pui Wang from Department of Microbiology, the University of Hong Kong, for providing the A549 cell line. We would also like to express our gratitude to Prof. Zhiwu Tan from Department of Applied Biology and Chemical Technology, Faculty of Science, the Hong Kong Polytechnic University, for providing the AB1 mesothelioma tumor cell line.

Z.C. reports grants from Theme-Based Research Scheme (T11-702/24-N) of the Hong Kong Research Grants Council, Seed Fund for Translational and Applied Research of University Research Committee (URC) of HKU, and the Collaborative Research with GeneHarbor (Hong Kong) Biotechnologies Limited; University Development Fund and Li Ka Shing Faculty of Medicine Matching Fund from HKU to AIDS Institute.

AUTHOR CONTRIBUTIONS

H.X., conceptualization, data curation, software, formal analysis, methodology, and writing – review and editing draft; M.C.T.W., software, formal analysis, and methodology; Y.W., S.Q., and C.C.C.W., methodology; J.W. and Z.C., conceptualization, supervision, and writing – review and editing.

DECLARATION OF INTERESTS

J.W. is an employee and shareholder of GeneHarbor (Hong Kong) Biotechnologies.

SUPPLEMENTAL INFORMATION

Supplemental information can be found online at https://doi.org/10.1016/j.omton.2026.201221.

REFERENCES

1. Wang, T., Lee, S., and Feldman, G.M. (2020). Immunotherapy With Programmed Cell Death 1 vs Programmed Cell Death Ligand 1 Inhibitors in Patients With Cancer. JAMA Oncol. *6*, 1115. https://doi.org/10.1001/jamaoncol.2020.0631.
2. Pardoll, D.M. (2012). The blockade of immune checkpoints in cancer immunotherapy. Nat. Rev. Cancer *12*, 252–264. https://doi.org/10.1038/nrc3239.
3. Cha, J.H., Chan, L.C., Li, C.W., Hsu, J.L., and Hung, M.C. (2019). Mechanisms Controlling PD-L1 Expression in Cancer. Mol. Cell *76*, 359–370. https://doi.org/10.1016/j.molcel.2019.09.030.
4. Klement, J.D., Redd, P.S., Lu, C., Merting, A.D., Poschel, D.B., Yang, D., Savage, N.M., Zhou, G., Munn, D.H., Fallon, P.G., and Liu, K. (2023). Tumor PD-L1 engages myeloid PD-1 to suppress type I interferon to impair cytotoxic T lymphocyte recruitment. Cancer Cell *41*, 620–636.e9. https://doi.org/10.1016/j.ccell.2023.02.005.
5. Belenky, P., Bogan, K.L., and Brenner, C. (2007). NAD+ metabolism in health and disease. Trends Biochem. Sci. *32*, 12–19. https://doi.org/10.1016/j.tibs.2006.11.006.
6. Yoshino, J., Baur, J.A., and Imai, S.I. (2018). NAD(+) Intermediates: The Biology and Therapeutic Potential of NMN and NR. Cell Metab. *27*, 513–528. https://doi.org/10.1016/j.cmet.2017.11.002.
7. Mo, Y., Yue, M., Yim, L.Y., Zhou, R., Yu, C., Peng, Q., Zhou, Y., Luk, T.Y., Lui, G.C.Y., Huang, H., et al. (2023). Nicotinamide mononucleotide impacts HIV-1 infection by modulating immune activation in T lymphocytes and humanized mice. EBioMedicine *98*, 104877. https://doi.org/10.1016/j.ebiom.2023.104877.
8. Sun, Q., Hong, Z., Zhang, C., Wang, L., Han, Z., and Ma, D. (2023). Immune checkpoint therapy for solid tumours: clinical dilemmas and future trends. Signal Transduct. Target. Ther. *8*, 320. https://doi.org/10.1038/s41392-023-01522-4.
9. Lu, M.J., Busquets, J., Impedovo, V., Wilson, C.N., Chan, H.R., Chang, Y.T., Matsui, W., Tiziani, S., and Cambronne, X.A. (2024). SLC25A51 decouples the mitochondrial NAD(+)/NADH ratio to control proliferation of AML cells. Cell Metab. *36*, 808–821.e6. https://doi.org/10.1016/j.cmet.2024.01.013.
10. Xu, H., Yue, M., Zhou, R., Wang, P., Wong, M.Y.C., Wang, J., Huang, H., Chen, B., Mo, Y., Tam, R.C.Y., et al. (2024). A Prime-Boost Vaccination Approach Induces Lung Resident Memory CD8+ T Cells Derived from Central Memory T Cells That Prevent Tumor Lung Metastasis. Cancer Res. *84*, 3173–3188. https://doi.org/10.1158/0008-5472.CAN-23-3257.
11. Alley, E.W., Lopez, J., Santoro, A., Morosky, A., Saraf, S., Piperdi, B., and van Brummelen, E. (2017). Clinical safety and activity of pembrolizumab in patients with malignant pleural mesothelioma (KEYNOTE-028): preliminary results from a non-randomised, open-label, phase 1b trial. Lancet Oncol. *18*, 623–630. https://doi.org/10.1016/S1470-2045(17)30169-9.
12. Tan, Z., Chiu, M.S., Yan, C.W., Wong, Y.C., Huang, H., Man, K., and Chen, Z. (2020). Antimesothelioma Immunotherapy by CTLA-4 Blockade Depends on Active PD1-Based TWIST1 Vaccination. Mol. Ther. Oncolytics *16*, 302–317. https://doi.org/10.1016/j.omto.2020.01.009.
13. Schunselaar, L.M., Quispel-Janssen, J.M.M.F., Neefjes, J.J.C., and Baas, P. (2016). A catalogue of treatment and technologies for malignant pleural mesothelioma. Expert Rev. Anticancer Ther. *16*, 455–463. https://doi.org/10.1586/14737140.2016.1162100.
14. Geels, S.N., Moshensky, A., Sousa, R.S., Murat, C., Bustos, M.A., Walker, B.L., Singh, R., Harbour, S.N., Gutierrez, G., Hwang, M., et al. (2024). Interruption of the intratumor CD8(+) T cell:Treg crosstalk improves the efficacy of PD-1 immunotherapy. Cancer Cell *42*, 1051–1066.e7. https://doi.org/10.1016/j.ccell.2024.05.013.
15. Huang, Q., Wu, X., Wang, Z., Chen, X., Wang, L., Lu, Y., Xiong, D., Liu, Q., Tian, Y., Lin, H., et al. (2022). The primordial differentiation of tumor-specific memory CD8(+) T cells as bona fide responders to PD-1/PD-L1 blockade in draining lymph nodes. Cell *185*, 4049–4066.e25. https://doi.org/10.1016/j.cell.2022.09.020.

16. Anderson, G.M., Nakada, M.T., and DeWitte, M. (2004). Tumor necrosis factor-alpha in the pathogenesis and treatment of cancer. Curr. Opin. Pharmacol. *4*, 314–320. https://doi.org/10.1016/j.coph.2004.04.004.

17. Buscher, K., Ehinger, E., Gupta, P., Pramod, A.B., Wolf, D., Tweet, G., Pan, C., Mills, C.D., Lusis, A.J., and Ley, K. (2017). Natural variation of macrophage activation as disease-relevant phenotype predictive of inflammation and cancer survival. Nat. Commun. *8*, 16041. https://doi.org/10.1038/ncomms16041.

18. Li, W., Li, Y., Jin, X., Liao, Q., Chen, Z., Peng, H., and Zhou, Y. (2022). CD38: A Significant Regulator of Macrophage Function. Front. Oncol. *12*, 775649. https://doi.org/10.3389/fonc.2022.775649.

19. Stein, L.R., and Imai, S.i. (2012). The dynamic regulation of NAD metabolism in mitochondria. Trends Endocrinol. Metab. *23*, 420–428. https://doi.org/10.1016/j.tem.2012.06.005.

20. Ryu, K.W., Nandu, T., Kim, J., Challa, S., DeBerardinis, R.J., and Kraus, W.L. (2018). Metabolic regulation of transcription through compartmentalized NAD(+) biosynthesis. Science *360*, eaan5780. https://doi.org/10.1126/science.aan5780.

21. Yu, B., Jing, X., Jia, L., Wang, M., Liu, L., Ping, S., Wang, Y., and Yang, M. (2024). The versatile multi-functional substance NMN: its unique characteristics, metabolic properties, pharmacodynamic effects, clinical trials, and diverse applications. Front. Pharmacol. *15*, 1436597. https://doi.org/10.3389/fphar.2024.1436597.

22. De Palma, M., and Lewis, C.E. (2013). Macrophage regulation of tumor responses to anticancer therapies. Cancer Cell *23*, 277–286. https://doi.org/10.1016/j.ccr.2013.02.013.

23. Virassamy, B., Caramia, F., Savas, P., Sant, S., Wang, J., Christo, S.N., Byrne, A., Clarke, K., Brown, E., Teo, Z.L., et al. (2023). Intratumoral CD8(+) T cells with a tissue-resident memory phenotype mediate local immunity and immune checkpoint responses in breast cancer. Cancer Cell *41*, 585–601.e8. https://doi.org/10.1016/j.ccell.2023.01.004.

OMTON, Volume *34*

Supplemental information

Nicotinamide mononucleotide enhances anti-tumor effect by resetting macrophages toward the inflammatory M1-like phenotype

Haoran Xu, Marcus Chun Tao Wan, Chelsey Chi Ching Wong, Siqi Qin, Yang Wen, Jun Wang, and Zhiwei Chen

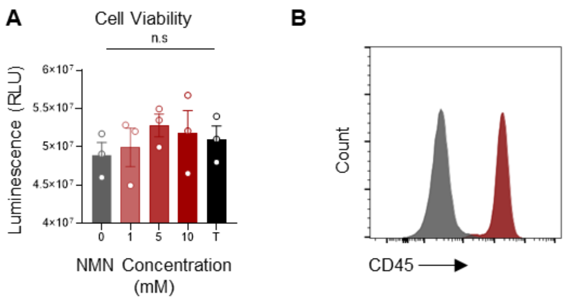

Figure S1. Experimental setup for huPBMC-tumor co-culture system. (**A**) The viability of tumor cells attached to the plate was determined by CellTiter-Glo® luminescent cell viability assay (Promega) at 24 hours post-co-culture with immune cells. The co-culture assay was set up following the same conditions as in Figure 1, and tumor cell only (T) served as the positive control (100% live cells) for viability measurement. (**B**) Cells harvested from the supernatant of three 10mM NMN-treated huPBMC-tumor coculture samples were combined and proceeded to the FACS for cell type analysis. All cells were divided into two samples for isotype control staining (grey) and CD45^{+} staining (red). Statistics in **A** were generated by one-way ANOVA with post-hoc correction followed by multiple comparisons.

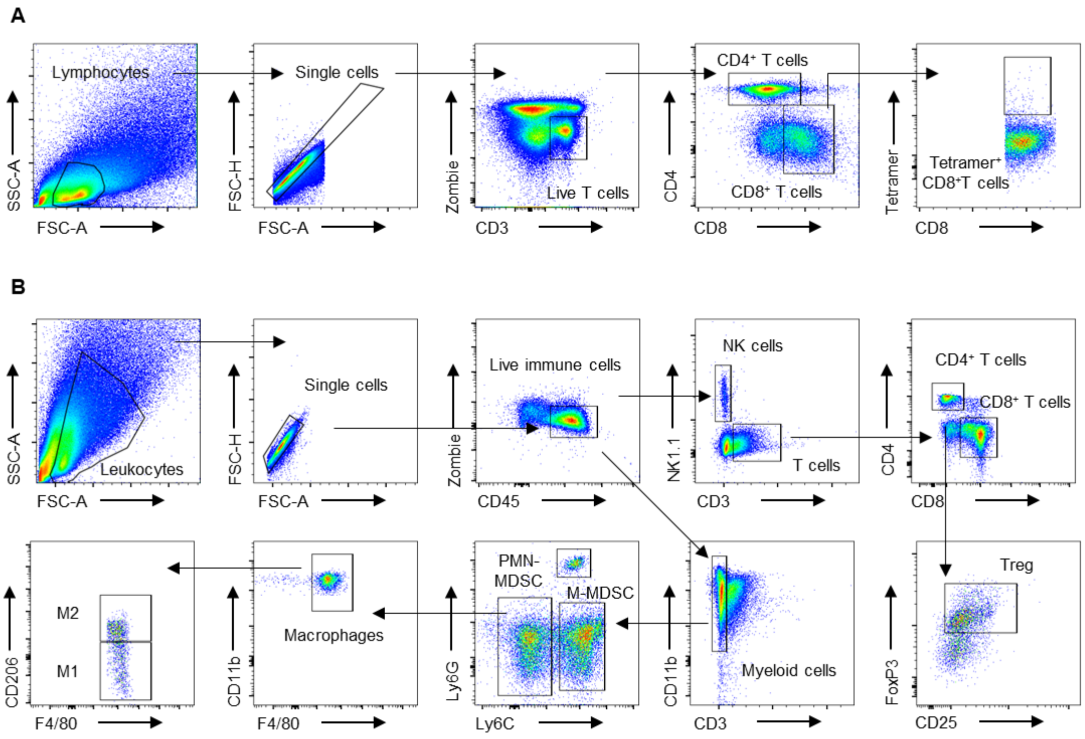

Figure S2. FACS Gating strategy. (**A**) Gating strategy for tumor-specific CD8^{+} T cells in spleen. (**B**) Gating strategy for intratumor immune cells.

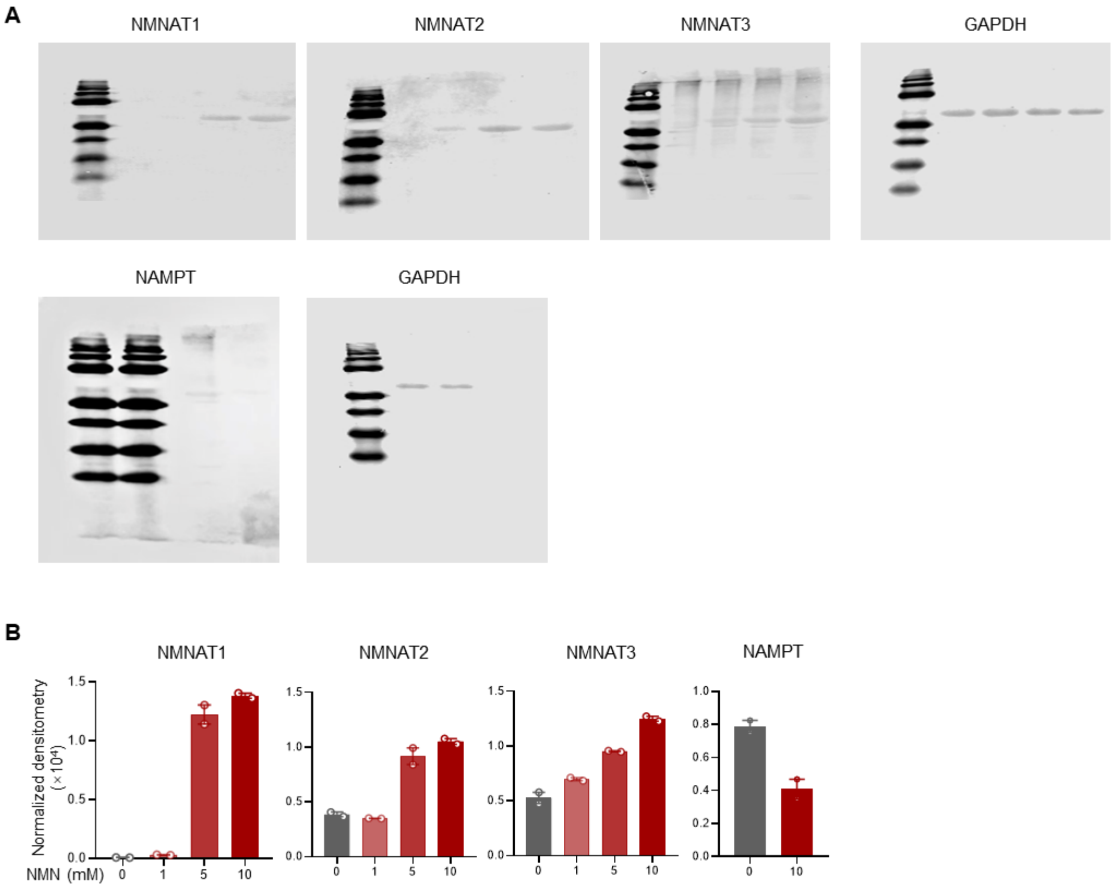

Figure S3. Original Western Blot data and densitometry quantification. (**A**) Matched original Western Blot data of Figure 1E and F (**B**) Densitometry quantification results from two independent experiments.

Table S1. List of primers for RT-qPCR.

Primer pair	Forward/ Reverse	Target sequence (5' → 3')
CD38 (mouse)	Forward	TCTCTAGGAAAGCCCAGATCG
	Reverse	AGAAAAGTGCTTCGTGGTAGG
GAPDH (human)	Forward	GGAGCGAGATCCCTCCAAAAT
	Reverse	GGCTGTTGTCATACTTCTCATGG
GAPDH (mouse)	Forward	AGGTCGGTGTGAACGGATTTG
	Reverse	GGGGTCGTTGATGGCAACA
IL-12 β (mouse)	Forward	GTCCTCAGAAGCTAACCATCTCC
	Reverse	CCAGAGCCTATGACTCCATGTC
NAMPT (human)	Forward	CGGCAGAAGCCGAGTTCAA
	Reverse	GCTTGTGTTGGGTGGATATTGTT
NMNAT1 (human)	Forward	TCTCCTTGCTTGTGGTTCATTC
	Reverse	TGACAACTGTGTACCTTCCTGTT
NMNAT2 (human)	Forward	TGTCCACGACTCCTATGGAAA
	Reverse	GTCCGATCACAGGTGTCATGG
NMNAT3 (human)	Forward	GAGTAGGTCACGACCCAAAAG
	Reverse	TCGCCTGATGTATGTGGCAC
Slc12a8 (human)	Forward	CTGGTGTCCTTCGTCATCCTG
	Reverse	CACCTGCAACACACTGTCCA
TNFa (mouse)	Forward	CAGGCGGTGCCTATGTCTC
	Reverse	CGATCACCCCGAAGTTCAGTAG
